# White, affluent, educated parents are least likely to choose HPV vaccination for their children: a cross-sectional study of the National Immunization Study – teen

**DOI:** 10.1186/s12887-017-0953-2

**Published:** 2017-12-01

**Authors:** Echo L. Warner, Qian Ding, Lisa M. Pappas, Kevin Henry, Deanna Kepka

**Affiliations:** 1 Cancer Control and Population Sciences, Huntsman Cancer Institute, University of Utah, 2000 Circle of Hope, Salt Lake City, UT 84112 USA; 2 Study Design and Biostatistics Center, School of Medicine, University of Utah, 295 Chipeta Way, Salt Lake City, UT 84123 USA; 3 Department of Geography, Temple University, 1115 Polett Walk, Philadelphia, PA 19112 USA; 4 Fox Chase Cancer Center, Cancer Prevention and Control Program, 333 Cottman Avenue, Philadelphia, Pennsylvania, 19111 USA; 5 College of Nursing, University of Utah, 10 S 2000 E, Salt Lake City, UT 84112 USA; 6 Biostatistics Center, Huntsman Cancer Institute, 2000 Circle of Hope, Salt Lake City, UT 84112 USA

**Keywords:** Adolescent, Ethnicity, Gender, HPV, Males

## Abstract

**Background:**

Human Papillomavirus (HPV) vaccination coverage is below national goals in the United States. Research is needed to inform strategically designed interventions that target sociodemographic groups with underutilization of HPV vaccination.

**Methods:**

Secondary data analysis of the National Immunization Survey-Teen 2013 measured association of sociodemographic factors (e.g., ethnicity/race, insurance) with HPV vaccination among females and males ages 13–17 (*N* = 18,959). Chi-square and multivariable Poisson regressions were conducted using survey-weighted statistics.

**Results:**

Having a mother ≥35 years, a mother with some college, being of “Other” ethnicity/race, and having no providers who order vaccines from health departments was negatively associated with females initiating HPV vaccination. Having a mother with some college, being of Non-Hispanic White or “Other” ethnicity/race, and having some or no providers who order vaccines from health departments was negatively associated with males initiating HPV vaccination. These same factors were negatively associated with males completing HPV vaccination with the exception of “Other” ethnicity/race. In contrast, having an unmarried mother, being ages 15–17, having a hospital based provider, and receiving other adolescent vaccinations were positively associated with females initiating and completing HPV vaccination. Having an unmarried mother, health insurance that is not employer or union sponsored, and influenza and meningitis vaccinations was positively associated with male’s initiating HPV vaccination. For males, being 15 or 17 years old and having other adolescent vaccinations was positively associated with vaccine completion. All findings *p* ≤ 0.05.

**Conclusions:**

Future HPV vaccination interventions may benefit from targeting certain sociodemographic groups that were negatively associated with HPV vaccination in this study.

## Background

Vaccination for Human Papillomavirus (HPV) is below Healthy People 2020 goals of 80% completion (three doses) among adolescents in the United States (U.S.) [1]. While missed opportunities for HPV vaccination and provider recommendation of the HPV vaccine influence parents’ and adolescents’ decisions to vaccinate [2, 3], other sociodemographic factors (e.g., age, ethnicity, insurance status etc.) play a key role in identifying groups of individuals who are least likely to receive the HPV vaccine.

Multiple systematic reviews have been completed on HPV vaccination and sociodemographic factors that are associated with HPV vaccination. A review of HPV beliefs and acceptability of the HPV vaccine summarized that parents with lower education are more accepting of the HPV vaccine, but presented mixed findings on the influence of insurance status, educational level, ethnicity/race, and household income on HPV vaccination [4]. Another review of the literature identified barriers to HPV vaccination among healthcare providers (e.g., providing only risk-based recommendations, financial challenges including cost to parents and lack of insurance reimbursement), parents and caregivers (e.g., lack of information or provider recommendation, concerns about cost and side effects), and underserved populations (e.g., limited information, being uninsured, low completion of series) [5]. These summaries of the literature provide context for studying and interpreting associations of sociodemographic factors with HPV vaccination using a national dataset of adolescent immunizations, the National Immunization Survey-Teen (NIS-Teen).

In the U.S., the NIS-Teen has been previously used to study relationships between sociodemographic factors and HPV vaccination patterns [6–10]. One study analyzing NIS-Teen data spanning 2008–2011 found that HPV vaccination patterns differ from other adolescent vaccinations in that below-poverty adolescents and minority race/ethnicity adolescents had higher series initiation compared to above-poverty and white adolescents [6]. In addition to poverty status and race/ethnicity, there are other social factors that have been associated with HPV vaccination including older adolescent age, being seen in public or hospital facilities, and having received other adolescent vaccinations [4, 10, 11].

Furthermore, gender differences exist in the HPV vaccination literature, with males being less likely to know about HPV and the HPV vaccine [12–14], and less likely to receive the HPV vaccine compared to females. In 2013, only 6.8% of boys completed three doses of the HPV vaccine, compared to 33.4% girls [15]. It is possible that differential initiation and completion of the HPV vaccine by gender may be due to the HPV vaccine being recommended for the first time in 2006 for girls and later for boys in 2011 [16, 17]. However, existing literature suggests that HPV vaccination is lower for boys because parents are unaware that boys can receive and benefit from HPV vaccines, both parents and providers prefer to vaccinate females over males, and concerns about costs [5, 14].

The primary objective of this study was to identify sociodemographic factors of individuals who are least likely to initiate and complete HPV vaccination. We hypothesized that younger adolescents would be less likely to be vaccinated than older adolescents, and that adolescents who had received other vaccinations would be more likely to be vaccinated compared to adolescents who had not received other vaccinations. We perform separate analyses to evaluate associations of sociodemographic factors with HPV vaccination for girls and boys based on the previously mentioned literature of differential HPV vaccination by gender. This study expands prior research by using the NIS-Teen to determine whether patterns of sociodemographic factors that have previously been associated with HPV vaccination persist in a more recent national NIS-Teen survey. To our knowledge, this study is the first to determine associations between sociodemographic factors with HPV vaccination using NIS-Teen data collected in 2013.

## Methods

### Study design and setting

A secondary cross-sectional data analysis of NIS-Teen 2013 data was performed to measure the association of sociodemographic factors with HPV vaccination among adolescents ages 13–17. The NIS-Teen is a publicly available, nationally representative survey with a complex sampling design [18]. Annually, the NIS-Teen surveys parents (telephone) and adolescent healthcare providers (mailed). In 2013, NIS-Teen household response rates were: cellular (23.3%) and landline (51.1%) [19]. The 2013 NIS-Teen sample is documented elsewhere, including the number of participants screened at each stage of the study and reasons for exclusion and nonresponse [19]. Analysis of publicly available data is considered exempt by the University of Utah Institutional Review Board.

### Consent for publication

Not applicable.

### Participants and sample size

Parents consented to have their adolescent’s provider contacted to verify vaccine receipt [19, 20]. While 68.3% of landline and 65.0% of cellular respondents agreed to have their adolescent’s provider contacted, only a total of 55.8% of all respodnents had sufficient provider-verified vaccination records to be included in the study [19]. Reasons for inadequate provider data included lack of parent/guardian consent to contact their adolescent’s provider, provider non-response, or inadequate information to contact providers [19]. Records with provider-verified immunization records from NIS-Teen 2013 were included (*N* = 18,959).

### Outcome variables

Outcomes included provider verified initiation (≥1 dose of the HPV vaccine) and completion (3 doses of the HPV vaccine) of the HPV vaccine. Variable weights adjust for respondents with missing provider data.

### Sociodemographic variables

The Social Ecological Framework (SEF) is a five-level framework of influence comprising: individual, interpersonal, organizational, community, and public policy factors [21]. Individual (e.g., teen’s age, poverty, ethnicity, and vaccination status), interpersonal (e.g., mother’s age, education, and marital status), and organizational (e.g., facility type for teen’s providers and provider’s vaccination ordering history) levels of influence were assessed herein. Marital status “other” category includes: never married, widowed, divorced, separated, and deceased. Ethnicity/race of teens “other” category includes: Non-Hispanic Black, Non-Hispanic Other, and Multiple Race. Groupings of sociodemographic variables were selected were selected a priori based on clinical relevance, existing literature, and prior research using NIS-Teen data [7, 8, 10].

### Statistical analysis

Provider-phase sampling weights were used to produce dual-frame point estimates and corresponding 95% confidence intervals (CI). Listwise deletion was used to handle records with missing values. Frequency counts and survey-weighted percentages were reported for gender subgroups separately to minimize bias. For both unordered and ordered categorical variables, so that even a nonlinear association could be detected, a survey weighted Pearson chi-square test was used to compare distributions of sociodemographic variables between those who initiated and completed HPV vaccination to those who did not. Survey weighted multivariable Poisson regressions were fitted to assess the association of selected sociodemographic variables, reported as adjusted prevalence ratios (PR) with 95% CI. Sociodemographic variables were assessed for multicollinearity and all variables were maintained in the final models. All tests were two-sided comparisons in STATA version 14.0; *p* < 0.05 were considered significant.

## Results

### Participants, sociodemographics, and HPV vaccination

Most mothers had at least high school education and were married. Adolescents were primarily living above poverty level, Non-Hispanic White, and on private health insurance. Univariate analyses indicated sociodemographic associations between HPV vaccine initiation and completion for females (Table 1): mother’s education, marital status (initiation only), poverty status, and teen’s ethnicity/race, age, providers’ facility type, providers ordering vaccines from state/local health departments (initiation only), and other recommended adolescent vaccinations (i.e., influenza, TDAP, Meningitis), all *p* < 0.05. For males: mother’s education, poverty status (initiation only), marital status (initiation only), and teen’s ethnicity/race, source of health insurance, providers ordering vaccines from state/local health departments (initiation only), and other recommended adolescent vaccinations (i.e., influenza, TDAP, Meningitis), all p < 0.05.

**Table 1:** Adolescent and parental sociodemographic characteristics, ^a^ NIS-Teen 2013

FEMALES				
Characteristic	≥1 dose of HPV vaccine		HPV vaccine completion^c^	
	No (*N* = 3776)	Yes (N = 5098)	No (*N* = 5484)	Yes (N = 3390)
	N (%)^b^	N (%)^b^	N (%)^b^	N (%)^b^
Age (Mother/Parent)				
≤34 years old	291 (37.1)	497 (62.9)	512 (64.4)	276 (35.6)
35–44 years old	1643 (44.2)	2048 (55.8)	2373 (64.3)	1318 (35.7)
≥ 45 years old	1842 (42.5)	2553 (57.5)	2599 (60.1)	1796 (39.9)
Education (Mother)				
< High school	329 (28.9)	629 (71.1)	567 (55.1)	391 (45.0)
High school	637 (40.8)	925 (59.2)	959 (60.1)	603 (40.0)
Some college	1123 (48.1)	1328 (51.9)	1585 (67.1)	866 (32.9)
College graduate	1687 (45.2)	2216 (54.8)	2373 (63.3)	1530 (36.7)
Poverty status				
Above poverty (>$75 k)	1716 (44.7)	2203 (55.4)	2376 (61.5)	1543 (38.5)
Above poverty (<=$75 k)	1469 (46.2)	1765 (53.8)	2118 (65.7)	1116 (34.4)
Below poverty	505 (33.2)	1009 (66.8)	863 (58.5)	651 (41.5)
Missing, n (%)	207 (2.33)		207 (2.33)	
Marital status of mother				
Married	2838 (45.5)	3483 (54.5)	3957 (63.0)	2364 (37.0)
Other^d^	938 (37.4)	1615 (62.6)	1527 (61.3)	1026 (38.7)
Ethnicity/Race of teens				
Hispanic	427 (32.5)	862 (67.5)	732 (55.2)	557 (44.8)
Non-Hispanic White	2642 (46.9)	3135 (53.1)	3645 (65.1)	2132 (34.9)
Other^d^	707 (41.9)	1101 (58.1)	1107 (62.3)	701 (37.7)
Age in years of selected teen				
13 years old	941 (49.4)	901 (50.6)	1381 (74.2)	452 (25.8)
14 years old	847 (44.9)	1020 (55.1)	1255 (67.9)	608 (32.1)
15 years old	738 (41.2)	1082 (58.8)	1079 (60.6)	729 (39.5)
16 years old	701 (40.0)	1147 (60.0)	1004 (56.9)	828 (43.1)
17 years old	549 (37.7)	1001 (62.3)	765 (51.8)	773 (48.2)
Source of health insurance for teens				
Provided through employment or union	2540 (44.3)	3198 (55.7)	3553 (62.1)	2185 (38.0)
Not Provided through employment or union	1187 (40.6)	1834 (59.4)	1858 (63.0)	1163 (37.0)
Missing, n (%)	115 (1.30)		115 (1.30)	
Facility type for teen’s providers				
All public facilities	592 (43.4)	710 (56.6)	897 (67.5)	405 (32.5)
All hospital facilities	317 (32.8)	530 (67.2)	459 (56.1)	388 (44.0)
All private facilities	1707 (42.6)	2296 (57.4)	2416 (60.2)	1587 (39.8)
Mixed/Other	1055 (45.1)	1486 (54.9)	1573 (64.7)	968 (35.3)
Missing, n (%)	181 (2.04)		181 (2.04)	
Do teen’s providers order vaccination from states/ local health department				
All providers	2487 (41.1)	3569 (58.9)	3683 (61.7)	2373 (38.2)
Some but possibly not all	511 (41.0)	735 (59.0)	768 (63.1)	478 (36.9)
No providers	453 (51.2)	444 (48.8)	590 (64.1)	307 (36.0)
Don’t know	278 (43.6)	350 (56.4)	396 (62.5)	232 (37.5)
Missing, n (%)	47 (0.53)		47 (0.53)	
Influenza vaccination^e^				
No	2697 (55.7)	2218 (44.3)	3594 (74.1)	1321 (25.9)
Yes	1079 (25.2)	2880 (74.8)	1890 (46.6)	2069 (53.4)
TDAP vaccination^f^				
No	1359 (54.0)	1067 (46.0)	1838 (75.8)	588 (24.2)
Yes	2417 (38.0)	4031 (62.0)	3646 (56.8)	2802 (43.2)
Meningitis vaccination^g^				
No	1558 (77.7)	558 (22.3)	1833 (90.4)	283 (9.6)
Yes	2218 (32.8)	4540 (67.2)	3651 (54.4)	3107 (45.6)
MALES				
Characteristic	≥1 dose of HPV vaccine		HPV vaccine completion^c^	
	No (*N* = 6522)	Yes (N = 3231)	No (*N* = 8375)	Yes (N = 1378)
	N (%)^b^	N (%)^b^	N (%)^b^	N (%)^b^
Age(Mother/Parent)				
≤ 34 years old	528 (59.6)	334 (40.4)	743 (85.8)	119 (14.2)
35–44 years old	2840 (64.7)	1301 (35.3)	3606 (87.4)	535 (12.6)
≥ 45 years old	3154 (67.3)	1596 (32.7)	4026 (84.9)	724 (15.1)
Education (Mother)				
< High school	622 (53.0)	464 (47.0)	898 (79.2)	188 (20.9)
High school	1182 (63.3)	574 (36.8)	1525 (86.2)	231 (13.8)
Some college	1912 (72.1)	770 (27.9)	2365 (88.9)	317 (11.1)
College graduate	2806 (66.9)	1423 (33.1)	3587 (86.8)	642 (13.2)
Poverty status				
Above poverty (>$75 k)	2919 (70.7)	1352 (29.3)	3649 (87.2)	622 (12.8)
Above poverty (<=$75 k)	2510 (67.8)	1064 (32.2)	3132 (86.9)	442 (13.1)
Below poverty	936 (53.3)	739 (46.7)	1384 (83.3)	291 (16.7)
Missing, n (%)	233 (2.39)		233 (2.39)	
Marital status of mother				
Married	4881 (68.6)	2184 (31.4)	6109 (86.7)	956 (13.3)
Other^d^	1641 (59.3)	1047 (40.7)	2266 (85.1)	422 (14.9)
Ethnicity/Race of teens				
Hispanic	814 (50.4)	657 (49.6)	1191 (79.7)	280 (20.3)
Non-Hispanic White only	4471 (73.3)	1835 (26.7)	5480 (88.9)	826 (11.1)
Other^d^	1237 (61.7)	739 (38.3)	1704 (86.1)	272 (13.9)
Age in years of selected teen				
13 years old	1361 (66.5)	614 (33.5)	1736 (88.3)	239 (11.7)
14 years old	1381 (64.9)	672 (35.1)	1777 (86.4)	276 (13.6)
15 years old	1262 (63.8)	652 (36.2)	1632 (84.7)	282 (15.4)
16 years old	1305 (64.1)	714 (35.9)	1702 (86.3)	317 (13.7)
17 years old	1213 (67.9)	579 (32.1)	1528 (84.9)	264 (15.1)
Source of health insurance for teens				
Provided through employment or union	4359 (70.6)	1885 (29.4)	5395 (87.5)	849 (12.5)
Not Provided through employment or union	2061 (58.6)	1297 (41.4)	2845 (84.4)	513 (15.6)
Missing, n (%)	151 (1.55)			
Facility type for teen’s providers				
All public facilities	984 (67.1)	421 (32.9)	1239 (87.7)	166 (12.3)
All hospital facilities	607 (61.6)	380 (38.4)	826 (84.5)	161 (15.5)
All private facilities	2973 (65.7)	1522 (34.3)	3829 (85.8)	666 (14.2)
Mixed/Other	1812 (65.3)	853 (34.7)	2299 (87.3)	366 (12.8)
Missing, n (%)	201 (2.06)		201 (2.06)	
Do teen’s providers order vaccination from states/ local health department				
All providers	4345 (62.8)	2410 (37.3)	5723 (84.9)	1032 (15.1)
Some but possibly not all	925 (67.6)	387 (32.5)	1158 (88.7)	154 (11.3)
No providers	745 (73.5)	225 (26.5)	871 (88.2)	99 (11.8)
Don’t know	458 (71.8)	209 (28.2)	574 (89.1)	93 (10.9)
Missing, n (%)	49 (0.50)		49 (0.50)	
Influenza vaccination^e^				
No	4263 (78.1)	1109 (21.9)	4976 (92.9)	396 (7.1)
Yes	2259 (48.7)	2122 (51.3)	3399 (77.2)	982 (22.8)
TDAP vaccination^f^				
No	2147 (76.3)	635 (23.7)	2561 (92.0)	221 (8.0)
Yes	4375 (61.2)	2596 (38.8)	5814 (83.9)	1157 (16.1)
Meningitis vaccination^g^				
No	2151 (92.8)	154 (7.2)	2249 (97.5)	56 (2.5)
Yes	4371 (57.5)	3077 (42.5)	6126 (82.9)	1322 (17.1)

^a^Adolescents with adequately complete provider-reported immunization records in the 2013 NIS-Teen survey were included in our analysis. Respondents from the U.S. Virgin Islands were excluded

^b^Unweighted frequencies and weighted percentages from Dual-Frame Sampling Weights

^c^HPV completion includes those who had received at least 3 doses of the HPV vaccine and is potentially overlapping with ≥1 dose of HPV vaccine

^d^Marital status “other” category includes: never married/widowed/divorced/separated/deceased. Ethnicity/race of teens “other” category includes: Non-Hispanic Black and Non-Hispanic Other & Multiple Race

^e^Adolescent has taken at least one dose of seasonal influenza vaccination in the past three years

^f^Adolescent has taken at least one dose of TDAP only vaccination since age 10 years old and before 13 years old

^g^Adolescent has taken at least one dose of Meningitis vaccination

Among female adolescents, 57.4% (*n* = 5098/8874) had received at least one dose of the HPV vaccine and 38.2% (*n* = 3390/8874) had received 3 doses of the HPV vaccine. In comparison, only 33.1% of male adolescents had received at least one dose of the HPV vaccine (*n* = 3231/9753), and 14.1% had received three doses of the HPV vaccine (*n* = 1378/9753).

### Females’ Sociodemographics and HPV initiation and completion

In Table 2, compared with mothers ≤34 years old, mothers aged 35–44 years and ≥45 years had lower prevalence of a daughter with HPV vaccine initiation (PR = 0.87, *p* ≤ 0.01). Mothers with some college had lower prevalence of a daughter who had initiated HPV vaccination than mothers with <High school education (PR = 0.90, *p* = 0.03). Unmarried mothers had higher prevalence of a daughter with HPV vaccine initiation (PR = 1.15, *p* < 0.01), and completion (PR = 1.12, *p* = 0.04) than married mothers. Compared to Hispanic female adolescents, females with Other race had lower prevalence of HPV vaccine initiation (PR = 0.91, *p* = 0.05). Female adolescents aged 15, 16, and 17 years had higher prevalence of HPV vaccine initiation (PR = 1.21–1.34, p < 0.01), and those aged 14, 15, 16, and 17 years had higher prevalence of completion (PR = 1.24–2.17, *p* ≤ .01) compared to 13-year olds. Female adolescents who saw providers in a hospital facility had higher prevalence of HPV vaccine initiation (PR = 1.11, *p* = 0.04) and completion (PR = 1.23, p = 0.05) than those who saw providers in public facilities. Female adolescents with no providers who utilized state/local health departments for vaccine supplies had lower prevalence of HPV vaccine initiation than those with all providers sourcing vaccine supplies from state/local health departments (PR = 0.89, *p* = 0.03). Female adolescents who had received seasonal influenza (PR = 1.46, *p* < 0.01), TDAP (PR = 1.09, *p* < 0.03), and meningitis (PR = 2.49, p < 0.01) vaccinations at recommended intervals had higher prevalence of initiating HPV vaccination as well as completing the HPV vaccine series (influenza: PR = 1.73, *p* < 0.01, TDAP: PR = 1.49, p < 0.01, and meningitis: PR = 3.35, p < 0.01) than those who were unvaccinated. Poverty status and type of health insurance for teens were not associated with HPV vaccination in multivariable analyses of female adolescents.

**Table 2:** Multivariable analysis of factors associated with HPV initiation and completion among female and male adolescents, NIS-Teen 2013^a^

FEMALES						
	≥1 dose of HPV vaccine (*N* = 8256)			HPV vaccine completion (N = 8256)		
	Adjusted vaccination coverage^b^ % (95%CI)	Adjusted prevalence ratio (95%CI)^c^	*p*-value	Adjusted vaccination coverage^b^ % (95%CI)	Adjusted prevalence ratio (95%CI)^c^	p-value
Age(Mother/Parent)						
≤ 34 years old	64.9 (59.1, 70.7)	Reference		41.7 (35.3, 48.1)	Reference	
35–44 years old	**56.4 (53.6, 59.1)**	**0.87 (0.79, 0.96)**	**<0.01**	36.8 (34.0, 39.6)	0.88 (0.75, 1.05)	0.15
≥ 45 years old	**56.8 (54.1, 59.4)**	**0.87 (0.79, 0.97)**	**0.01**	37.5 (34.8, 40.2)	0.90 (0.76, 1.07)	0.23
Education (Mother)						
< High school	61.7 (56.9, 66.5)	Reference		39.8 (33.2, 46.4)	Reference	
High school	59.1 (55.2, 62.9)	0.96 (0.87, 1.05)	0.35	41.6 (37.3, 45.9)	1.04 (0.87, 1.25)	0.63
Some college	**55.2 (51.7, 58.7)**	**0.90 (0.81, 0.99)**	**0.03**	34.6 (31.2, 37.9)	0.87 (0.72, 1.05)	0.15
College graduate	56.1 (52.9, 59.3)	0.91 (0.82, 1.01)	0.07	36.2 (33.0, 39.4)	0.91 (0.74, 1.11)	0.36
Poverty status						
Above poverty (>$75 k)	57.5 (54.0, 61.0)	Reference		37.9 (34.1, 41.6)	Reference	
Above poverty (<=$75 k)	55.4 (52.5, 58.4)	0.96 (0.89, 1.04)	0.35	36.5 (33.4, 39.5)	0.96 (0.85, 1.09)	0.55
Below poverty	60.1 (56.0, 64.3)	1.05 (0.94, 1.16)	0.39	38.7 (33.8, 43.6)	1.02 (0.85, 1.23)	0.82
Marital status of mother						
Married	54.5 (52.1, 56.8)	Reference		36.1 (33.7, 38.4)	Reference	
Other^d^	**62.7 (59.5, 65.8)**	**1.15 (1.08, 1.23)**	**<0.01**	**40.4 (36.9, 43.8)**	**1.12 (1.01, 1.25)**	**0.04**
Ethnicity/Race of teens						
Hispanic	61.1 (56.8, 65.4)	Reference		41.2 (36.1, 46.2)	Reference	
Non-Hispanic White only	56.5 (54.1, 59.0)	0.93 (0.85, 1.01)	0.07	36.4 (34.0, 38.7)	0.88 (0.77, 1.02)	0.09
Other^d^	**55.9 (52.4, 59.4)**	**0.91 (0.84, 1.00)**	**0.05**	37.0 (33.5, 40.5)	0.90 (0.78, 1.04)	0.16
Age in years of selected teen						
13 years old	48.7 (45.2, 52.2)	Reference		24.3 (21.0, 27.7)	Reference	
14 years old	53.0 (49.2, 56.7)	1.09 (0.99, 1.19)	0.08	**30.2 (26.7, 33.7)**	**1.24 (1.04, 1.48)**	**0.01**
15 years old	**59.0 (55.1, 62.8)**	**1.21 (1.10, 1.33)**	**<0.01**	**39.6 (35.5, 43.7)**	**1.63 (1.38, 1.92)**	**<0.01**
16 years old	**62.6 (58.7, 66.5)**	**1.28 (1.17, 1.41)**	**<0.01**	**45.4 (41.1, 49.6)**	**1.86 (1.58, 2.19)**	**<0.01**
17 years old	**65.1 (60.7, 69.5)**	**1.34 (1.21, 1.47)**	**<0.01**	**52.8 (48.0, 57.5)**	**2.17 (1.84, 2.55)**	**<0.01**
Source of health insurance for teens						
Provided through employment or union	57.0 (54.4, 59.5)	Reference		37.5 (34.9, 40.2)	Reference	
Not Provided through employment or union	58.0 (54.7, 61.2)	1.02 (0.94, 1.10)	0.65	37.6 (34.0, 41.1)	1.00 (0.88, 1.14)	0.99
Facility type for teen’s providers						
All public facilities	58.2 (53.8, 62.7)	Reference		34.0 (28.8, 39.3)	Reference	
All hospital facilities	**64.7 (59.7, 69.7)**	**1.11 (1.00, 1.23)**	**0.04**	**41.9 (35.7, 48.0)**	**1.23 (1.00, 1.51)**	**0.05**
All private facilities	57.1 (54.4, 59.9)	0.98 (0.90, 1.07)	0.67	38.6 (35.9, 41.3)	1.13 (0.96, 1.34)	0.14
Mixed/Other	55.0 (51.3, 58.6)	0.94 (0.86, 1.04)	0.25	35.9 (32.3, 39.6)	1.06 (0.88, 1.27)	0.57
Do teen’s providers order vaccination from states/ local health department						
All providers	57.8 (55.6, 60.0)	Reference		37.7 (35.5, 39.9)	Reference	
Some but possibly not all	60.3 (54.7, 65.9)	1.04 (0.94, 1.15)	0.39	37.4 (31.7, 43.0)	0.99 (0.84, 1.16)	0.91
No providers	**51.5 (46.1, 56.8)**	**0.89 (0.80, 0.99)**	**0.03**	37.5 (32.2, 42.8)	1.00 (0.86, 1.16)	0.95
Don’t know	57.5 (50.0, 64.9)	0.99 (0.87, 1.14)	0.94	36.8 (30.1, 43.6)	0.98 (0.81, 1.18)	0.81
Influenza vaccination^e^						
No	47.4 (44.8, 50.0)	Reference		28.1 (25.6, 30.6)	Reference	
Yes	**69.2 (66.8, 71.6)**	**1.46 (1.37, 1.55)**	**<0.01**	**48.5 (45.8, 51.3)**	**1.73 (1.56, 1.91)**	**<0.01**
TDAP vaccination^f^						
No	53.7 (49.7, 57.6)	Reference		27.5 (23.9, 31.1)	Reference	
Yes	**58.6 (56.5, 60.7)**	**1.09 (1.01, 1.18)**	**0.03**	**40.9 (38.7, 43.2)**	**1.49 (1.29, 1.72)**	**<0.01**
Meningitis vaccination^g^						
No	26.0 (22.3, 29.8)	Reference		12.7 (10.1, 15.3)	Reference	
Yes	**64.8 (62.7, 67.0)**	**2.49 (2.15, 2.89)**	**<0.01**	**42.6 (40.3, 44.8)**	**3.35 (2.71, 4.15)**	**<0.01**
MALES						
	≥1 dose of HPV vaccine (*N* = 9003)			HPV vaccine completion (N = 9003)		
	Adjusted vaccination coverage^b^ % (95%CI)	Adjusted prevalence ratio (95%CI)^c^	p-value	Adjusted vaccination coverage^b^ % (95%CI)	Adjusted prevalence ratio (95%CI)^c^	*p*-value
Age(Mother/Parent)						
≤ 34 years old	35.0 (30.3, 39.8)	Reference		12.9 (9.4, 16.5)	Reference	
35–44 years old	35.2 (32.5, 37.8)	1.00 (0.87, 1.16)	0.97	12.6 (10.6, 14.5)	0.97 (0.71, 1.33)	0.87
≥ 45 years old	33.4 (30.6, 36.2)	0.95 (0.81, 1.12)	0.57	14.9 (12.7, 17.1)	1.15 (0.83, 1.60)	0.40
Education (Mother)						
< High school	35.2 (30.4, 39.9)	Reference		18.2 (13.4, 23.1)	Reference	
High school	34.6 (30.8, 38.3)	0.98 (0.84, 1.14)	0.82	13.5 (10.4, 16.6)	0.74 (0.53, 1.03)	0.08
Some college	**29.6 (26.5, 32.7)**	**0.84 (0.71, 1.00)**	**0.05**	**11.4 (9.3, 13.5)**	**0.62 (0.45, 0.86)**	**<0.01**
College graduate	37.4 (33.7, 41.1)	1.06 (0.89, 1.28)	0.51	13.2 (11.1, 15.4)	0.73 (0.52, 1.02)	0.06
Poverty status						
Above poverty (>$75 k)	34.4 (30.6, 38.2)	Reference		14.8 (11.8, 17.9)	Reference	
Above poverty (<=$75 k)	32.7 (29.7, 35.7)	0.95 (0.83, 1.09)	0.47	13.1 (10.8, 15.4)	0.88 (0.70, 1.11)	0.30
Below poverty	36.5 (32.5, 40.6)	1.06 (0.88, 1.27)	0.52	13.0 (10.1, 16.0)	0.88 (0.61, 1.27)	0.49
Marital status of mother						
Married	32.7 (30.4, 35.1)	Reference		13.6 (11.9, 15.4)	Reference	
Other^d^	**37.2 (34.1, 40.4)**	**1.14 (1.02, 1.27)**	**0.02**	13.6 (11.4, 15.8)	1.00 (0.81, 1.23)	0.98
Ethnicity/Race of teens						
Hispanic	43.3 (38.8, 47.8)	Reference		16.2 (12.9, 19.4)	Reference	
Non-Hispanic White	**29.5 (27.3, 31.6)**	**0.68 (0.60, 0.77)**	**<0.01**	**12.3 (10.7, 13.8)**	**0.76 (0.60, 0.96)**	**0.02**
Other^d^	**35.7 (32.1, 39.4)**	**0.83 (0.72, 0.95)**	**<0.01**	13.9 (11.4, 16.5)	0.86 (0.66, 1.12)	0.26
Age in years of selected teen						
13 years old	32.0 (28.4, 35.6)	Reference		11.4 (8.8, 14.1)	Reference	
14 years old	33.0 (29.7, 36.3)	1.03 (0.90, 1.19)	0.67	11.5 (9.1, 13.9)	1.01 (0.74, 1.37)	0.96
15 years old	35.9 (31.8, 40.1)	1.12 (0.96, 1.31)	0.14	**15.9 (12.4, 19.3)**	**1.39 (1.01, 1.90)**	**0.04**
16 years old	36.1 (32.3, 39.8)	1.13 (0.97, 1.31)	0.11	12.9 (10.5, 15.3)	1.13 (0.84, 1.52)	0.41
17 years old	35.7 (31.5, 39.9)	1.12 (0.95, 1.31)	0.18	**17.5 (14.2, 20.8)**	**1.53 (1.13, 2.05)**	**<0.01**
Source of health insurance for teens						
Provided through employment or union	31.2 (28.7, 33.8)	Reference		12.6 (10.9, 14.3)	Reference	
Not Provided through employment or union	**38.3 (35.1, 41.5)**	**1.23 (1.08, 1.39)**	**<0.01**	15.0 (12.4, 17.6)	1.18 (0.93, 1.51)	0.17
Facility type for teen’s providers						
All public facilities	32.8 (27.9, 37.7)	Reference		13.5 (9.2, 17.8)	Reference	
All hospital facilities	37.0 (32.8, 41.2)	1.13 (0.94, 1.35)	0.19	14.6 (10.7, 18.5)	1.08 (0.72, 1.62)	0.71
All private facilities	33.4 (30.9, 36.0)	1.02 (0.87, 1.20)	0.82	13.2 (11.4, 15.0)	0.97 (0.69, 1.38)	0.88
Mixed/Other	36.5 (33.1, 40.0)	1.11 (0.94, 1.33)	0.22	14.4 (11.8, 17.1)	1.07 (0.74, 1.54)	0.72
Do teen’s providers order vaccination from states/ local health department						
All providers	36.1 (34.0, 38.3)	Reference		15.2 (13.5, 16.8)	Reference	
Some but possibly not all	**29.6 (24.6, 34.6)**	**0.82 (0.69, 0.98)**	**0.02**	**8.0 (5.6, 10.4)**	**0.53 (0.39, 0.72)**	**<0.01**
No providers	**29.5 (24.1, 35.0)**	**0.82 (0.68, 0.99)**	**0.04**	**10.7 (7.3, 14.1)**	**0.71 (0.51, 0.98)**	**0.04**
Don’t know	31.4 (24.5, 38.3)	0.87 (0.69, 1.09)	0.22	12.0 (8.0, 16.0)	0.79 (0.56, 1.12)	0.19
Influenza vaccination^e^						
No	24.5 (22.0, 26.9)	Reference		7.8 (6.3, 9.3)	Reference	
Yes	**44.9 (42.3, 47.6)**	**1.84 (1.64, 2.05)**	**<0.01**	**20.0 (17.9, 22.2)**	**2.58 (2.07, 3.20)**	**<0.01**
TDAP vaccination^f^						
No	31.4 (27.8, 35.0)	Reference		11.1 (8.6, 13.5)	Reference	
Yes	35.2 (33.1, 37.4)	1.12 (0.99, 1.27)	0.07	**14.3 (12.8, 15.9)**	**1.30 (1.01, 1.67)**	**0.04**
Meningitis vaccination^g^						
No	9.0 (6.4, 11.6)	Reference		4.0 (2.1, 5.9)	Reference	
Yes	**39.8 (37.7, 42.0)**	**4.43 (3.31, 5.93)**	**<0.01**	**15.4 (13.9, 16.9)**	**3.89 (2.39, 6.35)**	**<0.01**

^a^Bold indicates significance at p < 0.05, due to rounding some significant *p*-values are labeled 0.05

^b^Multivariable Poisson regression models are not based on an explicit causal structure and therefore the estimates may suffer from a “Table 2 fallacy” as described by Westreich and Greenland: The Table 2 Fallacy: Presenting and Interpreting Confounder and Modifier Coefficients. Am J Epidemiol. 2013;177(4):292–298

^c^Adjusted multivariable Poisson regressions estimate prevalence ratios by comparing one level of each variable to the reference level. Adjusted prevalence ratios are adjusted for all sociodemographic variables in the tables. Models were ran separately for females and males

^d^Marital status “other” category includes: never married/widowed/divorced/separated/deceased. Ethnicity/race of teens “other” category includes: Non-Hispanic Black and Non-Hispanic Other & Multiple Race

^e^Adolescent has taken at least one dose of seasonal influenza vaccination in the past three years

^f^Adolescent has taken at least one dose of TDAP only vaccination since age 10 years old and before 13 years old

^g^Adolescent has taken at least one dose of Meningitis vaccination

### Males’ Sociodemographics and HPV initiation and completion

In Table 2, mothers with some college had lower prevalence of a son initiating (PR = 0.84, *p* = 0.05) and completing (PR = 0.62, *p* < 0.01) HPV vaccination than those with <High school education. Unmarried mothers had higher prevalence of having a son initiate HPV vaccination than married mothers (PR = 1.14, *p* = 0.02). Compared with Hispanic male adolescents, Non-Hispanic White male adolescents (PR = 0.68, *p* < 0.01) and Other ethnicity/race male adolescents (PR = 0.83, p < 0.01) had lower prevalence of HPV vaccine initiation, and those of Non-Hispanic White ethnicity/race had lower HPV vaccine completion (PR = 0.76, p = 0.02) compared to Hispanic males. While age was not associated with initiation of the HPV vaccine, those who were 15 (PR = 1.39, *p* = 0.04) and 17 (PR = 1.53, *p* < 0.01) years old had higher prevalence of HPV vaccine completion compared to 13 year olds. Male adolescents with health insurance that was not provided through employment or union had higher prevalence of initiating HPV vaccination (PR = 1.23, *p* < 0.01) than those with employer or union sponsored health insurance. Male adolescents with some but possibly not all providers (PR = 0.82, *p* = 0.02) and none of providers (PR = 0.82, *p* = 0.04) utilizing state/local health departments for vaccine supplies had a lower prevalence of initiating and completing (PR = 0.53, *p* < 0.01, PR = 0.71, p = 0.04, respectively) HPV vaccination than those who reported all providers sourced vaccine supplies from state/local health department. Additionally, male adolescents who received seasonal influenza vaccination (PR = 1.84, *p* < 0.01) and meningitis vaccination (PR = 4.43, p < 0.01) at recommended timeframes had higher prevalence of HPV vaccine initiation than those who did not receive these vaccines. Male adolescents had higher prevalence of vaccine completion if they had received seasonal influenza vaccination (PR = 2.58, p < 0.01) TDAP (PR = 1.30, *p* = 0.04), and Meningitis vaccinations (PR = 3.89, p < 0.01) at recommended intervals. Mother’s age, poverty status, and facility type for teen’s providers were not associated with HPV vaccination in multivariable analyses of male adolescents.

We performed a Pearson correlation matrix of all sociodemographic variables to assess potential collinearity of variables included in the multivariable regression models. There was no evidence of multicollinearity so all variables were maintained in the final tables.

## Discussion

Sociodemographic factors like education level, marital and poverty status, and health insurance are considered social determinants of health because they influence the circumstances which determine an individual’s health and wellbeing [22]. By identifying sociodemographics that are associated with HPV vaccination, this research may inform future interventions in areas where social determinants are established (e.g., education systems, communities, healthcare organizations) to improve HPV vaccination [22]. Sociodemographic factors that were positively associated with HPV vaccination included not having employer/union sponsored health insurance, having an unmarried mother, and being of minority ethnicity/race. Other sociodemographic factors including mothers having some college education and being of non-Hispanic white ethnicity/race, were negatively associated with HPV vaccination.

Adolescents who are White, have married mothers, and who attend public facilities (girls) or state/local health departments (boys) were least likely to be vaccinated compared to their counterparts. These findings reflect previous research in that parents of White ethnicity/race and higher educated parents may be more hesitant about HPV vaccination [6, 23, 24], despite White individuals being more likely to have heard of the HPV vaccine compared to individuals of minority ethnicity [13]. Our finding of the negative association of married mothers with vaccination may, likewise, be a reflection of higher socioeconomic status and lower willingness to vaccinate. Clinical interventions in healthcare facilities that serve the public as well as state/local health departments are needed. Improving the delivery of strong HPV vaccination recommendations among clinicians who are employed in these facilities may improve parent’s willingness to vaccinate. There is an abundance of provider training resources for devising strong recommendations for HPV vaccination that could be implemented and tested in public facilities [25].

Girls with Medicaid reported significantly higher HPV vaccination completion compared to those with private insurance (19% vs. 12%) [26]. While HPV vaccination is available with no-cost sharing from most private health insurance plans, options for public financing (e.g., Medicaid, Vaccines for Children, Immunization Grant Program, and Children’s Health Insurance Program) for HPV vaccination may be more robust in comparison [27]. Moreover, differential provider recommendation may occur for those receiving services from providers who are covered under private versus public insurance plans. Reasons for differential receipt of the HPV vaccine between privately and publicly insured adolescents cannot be clearly determined without further research.

Receipt of adolescent vaccinations was positively associated with HPV vaccination. In 2013, the President’s Cancer Panel Report focused on improving HPV vaccination, and suggested three strategies to combat low vaccination coverage in the U.S. [28]. The first of these recommendations was to reduce missed clinical opportunities for HPV vaccination by coupling HPV vaccination with other adolescent immunizations [28]. Our finding of a strong association between meningitis vaccination and HPV vaccination is notable, and may be the result of bundled vaccinations and/or states requiring meningitis vaccination for school entry. Given the positive association between receipt of adolescent vaccinations and HPV vaccination, further research examining the feasibility of bundling HPV vaccination with other adolescent immunizations is needed and necessary for accelerating HPV vaccination in the U.S.

There are potential limitations to consider in the interpretation of these findings. Response bias may underrepresent individuals who declined to participate, and it is possible that nonresponders differ from participants on key sociodemographic characteristics, like income and race/ethnicity. The cross-sectional design of this data precludes our ability to assess changes in the association of sociodemographic factors over time. Furthermore, potential outcome misclassification of HPV vaccine completion may occur with cross-sectional surveys if an individual completes later doses of the HPV vaccine after the survey was conducted, but within the recommended time frame. However we performed a sensitivity analysis which indicated that all respondents who completed the HPV vaccine from our sample did so within the recommended 24 week timeframe. NIS-Teen survey weights do not resolve potential selection bias from limiting data to provider-validated records, which were unavailable for approximately 45% of the sample [19]. Although, provider validated records are generally similar in terms of sociodemographic characteristics compared to the full NIS-Teen sample, with the exception of having a larger proportion of married mothers and differential poverty status distribution. Lastly, although our results show statistically significant associations of sociodemographic factors with HPV vaccination, these associations may not demonstrate clinical significance.

## Conclusions

Sociodemographic factors that merit attention in the targeted design of HPV vaccination interventions within the three base levels of the SEF were evaluated. Future research may benefit from distinguishing between completion of two and three dose HPV vaccines and exploring community level geographic factors that may extend these findings due to the heterogeneity of vaccination by locale [29, 30]. Multifaceted, targeted interventions that consider sociodemographic variation are needed to improve HPV vaccination among sub-groups of adolescents in the U.S. Recent studies have shown protection from HPV infection with fewer than three doses of the HPV vaccine [31, 32], emphasizing the need to initiate HPV vaccination at a younger age. Targeted intervention efforts are needed to improve HPV vaccine receipt among boys, non-Hispanic whites, and more affluent parents.

## Abbreviations

CI: Confidence Interval
HPV: Human Papillomavirus
NIS-Teen: National Immunization Survey-Teen
PR: Prevalence Ratio
SEF: Social Ecological Framework
TDAP: Tetanus Diphtheria and Pertussis
US: United States
